# Blockade of ß-Adrenergic Receptors by Nebivolol Enables Tumor Control Potential for Uveal Melanoma in 3D Tumor Spheroids and 2D Cultures

**DOI:** 10.3390/ijms24065894

**Published:** 2023-03-20

**Authors:** Lina S. Farhoumand, Hongtao Liu, Theodora Tsimpaki, Ulrike B. Hendgen-Cotta, Tienush Rassaf, Nikolaos E. Bechrakis, Miltiadis Fiorentzis, Utta Berchner-Pfannschmidt

**Affiliations:** 1 Eye Research Lab, Department of Ophthalmology, University Hospital Essen, University of Duisburg-Essen, 45147 Essen, Germany; 2 CardioScience Labs, Department of Cardiology and Vascular Medicine, West German Heart and Vascular Center, University Hospital Essen, University of Duisburg-Essen, 45147 Essen, Germany

**Keywords:** ß1-selective blocker, nebivolol, uveal melanoma, 3D tumor spheroids, anti-tumor activity, tumor control potential

## Abstract

Uveal melanoma (UM) is the most common primary cancer of the eye in adults. A new systemic therapy is needed to reduce the high metastasis and mortality rate. As β-blockers are known to have anti-tumor effects on various cancer entities, this study focuses on investigating the effect of β1-selective blockers atenolol, celiprolol, bisoprolol, metoprolol, esmolol, betaxolol, and in particular, nebivolol on UM. The study was performed on 3D tumor spheroids as well as 2D cell cultures, testing tumor viability, morphological changes, long-term survival, and apoptosis. Flow cytometry revealed the presence of all three β-adrenoceptors with a dominance of β2-receptors on cell surfaces. Among the blockers tested, solely nebivolol concentration-dependently decreased viability and altered 3D tumor spheroid structure. Nebivolol blocked the repopulation of cells spreading from 3D tumor spheroids, indicating a tumor control potential at a concentration of ≥20 µM. Mechanistically, nebivolol induced ATP depletion and caspase-3/7 activity, indicating that mitochondria-dependent signaling is involved. D-nebivolol or nebivolol combined with the β2-antagonist ICI 118.551 displayed the highest anti-tumor effects, suggesting a contribution of both β1- and β2-receptors. Thus, the present study reveals the tumor control potential of nebivolol in UM, which may offer a perspective for co-adjuvant therapy to reduce recurrence or metastasis.

## 1. Introduction

Beta-adrenoceptor blockers are cardioprotective drugs used in various heart diseases due to their antagonistic action on the G-protein-coupled β-adrenergic receptors [1]. Furthermore, several studies revealed an anti-tumor effect of β-blockers, due to the regulation of various cellular processes that counteract tumor growth and metastasis, e.g., modulation of angiogenesis, cell proliferation, and cell survival [2,3]. These effects are attributed to different mechanisms, which are presumably also related to the respective tumor entity. Thus, non-selective β-blockers in cutaneous melanoma showed an effect via the activation of apoptosis, and studies on ovarian tumors proposed inhibition of macrophage recruitment by propranolol [4,5]. What they all have in common, however, is the modulation of stress and thus the tumor microenvironment, which occurs via the blocking of catecholamines by one of the three β-receptor subtypes [3,4,5]. Indeed, the majority of studies showing an anti-tumor effect of ß-adrenoceptor blockers are preclinical, yet β-receptors have already shown promising clinical effects in breast cancer, and propranolol, a non-selective ß-blocker, is already used as the gold standard in the active treatment of infantile hemangioma [3,6,7,8]. In the quest for potential novel targets and drugs to prevent tumor progression and metastasis, drug repurposing is of increasing interest. Thus, ß-blockers have advantages over new drugs as they have already undergone the time-consuming and costly process of the Food and Drug Administration (FDA) approval and thus represent an immediately available option, with the common contraindications and side effects already identified [9,10,11].

Beta-blockers can be classified into three different generations depending on their affinity for the ß1-receptor. First-generation β-blockers are non-selective and inhibit both β1- and β2-receptors. Second-generation β-blockers are more selective for β1-receptors, whereas third generation β-blockers are highly selective drugs for β1-receptors and have an additional vasodilatory property by acting on the α1- and β3-adrenoreceptors [1,12,13]. Due to the more selective inhibition of β1-receptors, the second and third generation β-blockers are associated with a lower side effect profile [1,8,12]. In particular, nebivolol, introduced by Van de Water et al. in 1988 [14], has a favorable side-effect profile in comparison with other β-blockers, as it does not lead to a worsening in patients with mild obstructive pulmonary disease or impotence respectively erectile dysfunction. Furthermore, a positive effect on glucose and lipid metabolism is discussed [15,16,17,18,19]. Nebivolol is a third generation β-blocker approved by the FDA in 2007 for the treatment of hypertension [16,20]. It depicts the highest selectivity for β1-receptors and has an additional vasodilatory effect mediated by interacting with the L-arginine/nitric oxide pathway via β3-receptor agonism, which may also lead to a reduction of oxidative stress [14,17,21,22]. A tumor inhibitory effect of nebivolol has recently been described in tumors such as breast, lung, and oral squamous cell cancer [11,23,24]. However, studies on the effect of β1-selective blockers, such as nebivolol, in ocular cancers are still lacking.

Uveal melanoma (UM), a tumor of the iris (4%), ciliary body (6%), and mostly the choroid (90%), represents the most common primary intraocular tumor in adults, with an incidence rate of approximately 5.7 in North America and an even higher incidence rate in Europe of 7.3 [25,26]. Therapy options range, depending on tumor size and location, from transpupillary thermo-therapy to radiation therapy, tumor resection, and as an ultimate option, enucleation [25,27]. UM leads to the development of metastases in approximately 50% of cases [28,29,30,31]. Once metastasis occurs, treatment options are limited, and mostly involve the liver as it is the first and most common site for metastases due to the spread of cells through the bloodstream. Interestingly, these often do not appear until several decades after the primary treatment of the tumor, which is due to an apparent dormancy of the cells and thus makes the therapy of UM even more difficult [29,31]. Other than local-regional treatments, intense effort has been made to find a systemic therapy; however, this has resulted in poor outcomes. [29,32,33]. Although UM, like its namesake cutaneous melanoma, arises from neoplastic melanocytes originating from the neural crest, UM differs biologically from cutaneous melanoma and, therefore, responds poorly to checkpoint inhibitors or chemotherapy [33,34].

Furthermore, the molecular pathogenesis of UM is characterized by other gene mutations and chromosomal abnormalities. By far the most common mutations are the guanine nucleotide-binding protein G(q) subunit α (GNAQ) and the guanine nucleotide-binding protein subunit alpha-11 (GNA11) mutation. These are also known as starting point mutations, which occur in approximately 85% of cases; however, they do not report a higher risk of metastases development [30,35]. Chromosomal abnormalities, such as 6p gain, are associated with a good prognosis, whereas BRCA1-associated protein1 (BAP1) gene mutation, 1p loss, 3 loss, and 8q gain are linked to poor outcomes. In particular, monosomy-3 is linked to the aggressive behavior of the tumor and is associated with a poor prognosis [25,36,37,38]. Additionally, prognosis is associated with histological characteristics, hence, the presence of the epithelioid cell type indicates a worse outcome than cells of the spindle type [25,39].

Recently, we and others have demonstrated the presence of β1- and β2-adrenergic receptors in UM tumor tissues and cell lines by using histochemistry [40,41]. Among the non-selective β-blockers, carvedilol, propranolol, and labetalol showed anti-tumor activity in UM cell lines [40,41]. In the present study, we aimed to identify the most effective anti-tumor ß-blocker and evaluated, for the first time, the anti-tumor effect of various β1-selective blockers in UM. Among all β1-blockers tested, nebivolol exclusively showed anti-tumor activity in UM 3D tumor spheroid models as well as in 2D cell cultures. We evaluated 3D tumor spheroid cell viability, apoptosis, morphological changes, as well as long-term cell survival. Our study, which included four different 3D tumor spheroid models and cell lines, suggests nebivolol and its enantiomers to be β1/β2-blockers with the potential for tumor control in UM.

## 2. Results

### 2.1. Identification of Nebivolol as a Potent Anti-Tumor β1-Blocker

**Figure 1:**
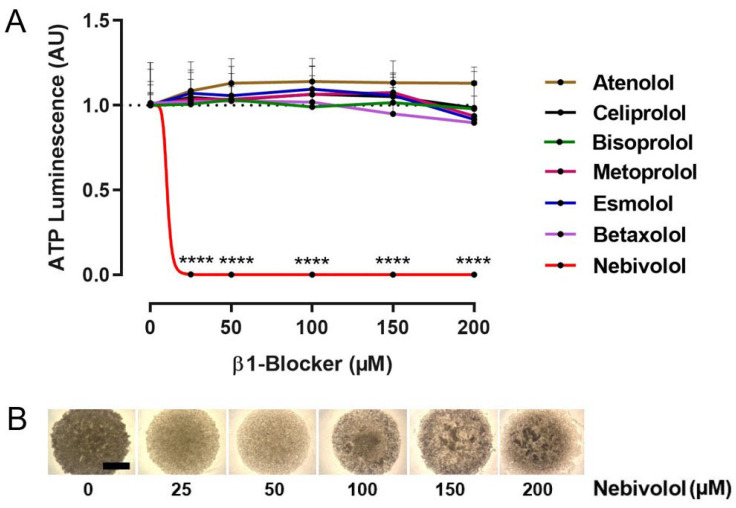
Screening for ß1-selective blockers with anti-tumor activity in 3D tumor spheroids. (**A**) Concentration-response curves of tumor spheroids generated from a Mel270 cell line. Spheroid viability was determined using an ATP luminescence assay, normalized to the ATP luminescence of the respective control spheroids (0 µM ß1-blocker), and is given in arbitrary units (AU). The means ± SD of at least three independent experiments for each β1-blocker with n = 6–8 spheroids for each concentration shown. Statistical analysis was performed using a one-way ANOVA and Tukey’s multiple comparisons test, and the significance levels of the treated spheroids in relation to the control spheroids (0 µM ß-blocker) are indicated at **** *p* < 0.0001. (**B**) Representative microscopic images of Mel270 tumor spheroids after 7-day incubation with nebivolol at the given concentrations (4× magnification), scale bars indicate 500 µm.

To identify β1-selective blockers with anti-tumor activity, we treated UM tumor spheroids with various β1-blockers in a concentration range of 0–200 µM and analyzed spheroid viability after 7 days (Figure 1). For this purpose, large and uniform tumor spheroids were generated from a Mel270 cell line. This cell line had been proven to be more therapy-resistant in a previous study, as it originated from a recurrent tumor after prior irradiation, and it was therefore used as a screening model cell line in the present study [42,43]. The ß1-blockers atenolol, celiprolol, bisoprolol, metoprolol, esmolol, and betaxolol had no significant effects on spheroid viability (Figure 1A). In contrast, nebivolol at ≥25 µM completely blocked spheroid viability (Figure 1A). Microscopic observation of the nebivolol treated spheroids revealed a concentration-dependent change in spheroid appearance, suggesting a reduction in size and compactness (Figure 1B).

### 2.2. Comparison of Nebivolol Anti-Tumor Responses of Various 3D Tumor Spheroids

**Figure 2:**
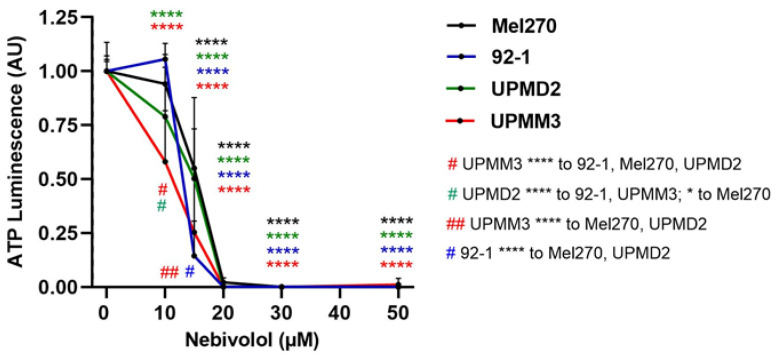
Effects of nebivolol on the viability of various 3D tumor spheroid models. Tumor spheroids were generated from cell lines 92-1 and Mel270 or primary cell lines UPMD2 and UPMM3, and were treated once with nebivolol at the indicated concentrations. Spheroid viability was determined using an ATP luminescence assay after 7 days of incubation with nebivolol. The luminescence of the treated spheroids was normalized to the luminescence of the control spheroids (0 µM nebivolol), and is given in arbitrary units (AU). Shown are the means ± SD of three independent experiments for each UM tumor spheroid type with n = 6–8 spheroids per condition. Statistical analysis was conducted using two-way ANOVA and Tukey’s multiple comparisons tests. The significance levels of treated spheroids relative to control spheroids (0 µM nebivolol) are shown in color. Significance levels are indicated at **** *p* < 0.0001. The significance level of nebivolol-treated spheroids relative to other spheroid types is indicated with # or ## (figure legend bottom right corner * *p* < 0.05, **** *p* < 0.0001).

Since nebivolol alone reduced 3D tumor spheroid viability, we next investigated the anti-tumor activity of nebivolol in more detail. Patient-derived UM cell lines differ genetically as well as in terms of cell morphology and proliferation rate, which may account for individual therapy responses. We hence generated tumor spheroids from another well-established cell line, 92-1, and two primary cell lines, UPMD2 and UPMM3, for comparison with the Mel270 spheroids. The viability of the spheroid types was diminished by nebivolol treatment in a concentration range of 10–30 µM (Figure 2). Among the spheroid types with disomy-3 genetic, the 92-1 spheroids were found to be most responsive to nebivolol treatment. The 92-1 spheroid viability was sharply reduced in response to ≥15 µM and blocked at 20 µM nebivolol, whereas the viability of the Mel270 spheroids was blocked at 30 µM nebivolol (Figure 2). The monosomy-3-containing UPMM3 spheroids were the most sensitive ones, as viability was significantly reduced at ≥10 µM and blocked at 20 µM nebivolol (Figure 2).

**Figure 3:**
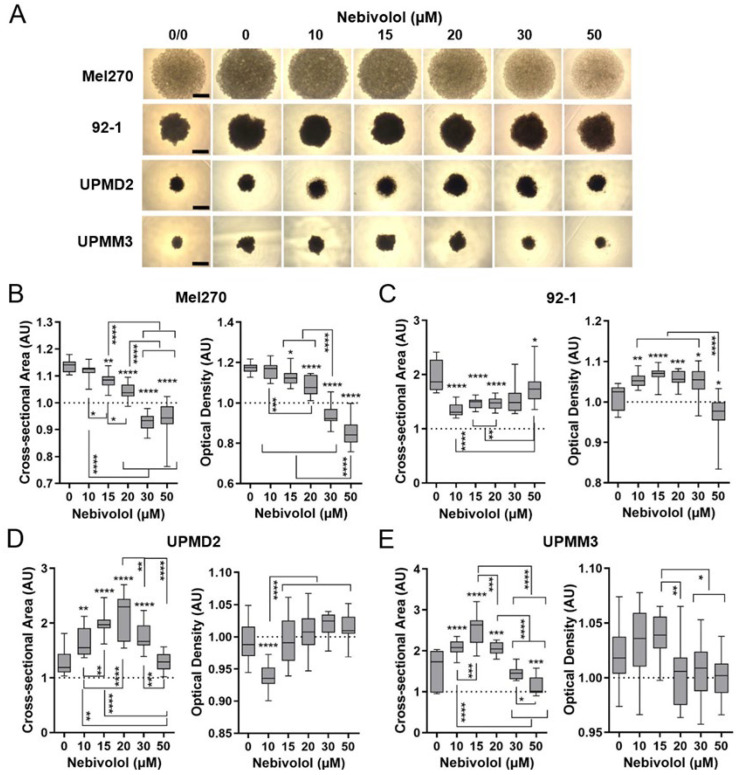
Morphology changes of 3D UM tumor spheroid types by nebivolol treatment. Mel270, 92-1, UPMD2, or UPMM3 derived spheroids were incubated with nebivolol at the indicated concentrations for 7 days. The spheroids were microscopically imaged before and after treatment, and cross-sectional area and density were measured. (**A**) Representative microscopic images of the spheroids treated with 0–50 µM nebivolol; untreated spheroids are marked 0/0. Spheroids were imaged at 4× magnification, scale bars indicate 500 µm. (**B**–**E**) Spheroid cross-sectional area and optical density of the respective spheroid types were determined and normalized to the respective spheroids before treatment (0/0). Box plots with min to max whiskers and a median of three independent experiments for each spheroid type with n = 5 spheroids each concentration shown. The means of the cross-sectional area or the optical density of the spheroids before treatment (0/0) are represented by dotted lines. A one-way ANOVA and Tukey’s multiple comparisons test were used for statistical analyses, significance levels are indicated at * *p* < 0.05, ** *p* < 0.01, *** *p* < 0.001, and **** *p* < 0.0001.

We next assessed the effects of nebivolol on the morphology of spheroid types and hence analyzed spheroid size and density of treated spheroids by microscopy (Figure 3). Drug concentrations ≥15 µM caused a significant decrease in the Mel270 spheroid area and density, indicating inhibition of spheroid growth and compactness (Figure 3A,B). Cell line 92-1 derived spheroids, however, only required drug concentrations of ≥10 µM for the reduction in size, while the density was increased, presumably caused by the shrinkage of the spheroids (Figure 3A,C). At a concentration of 50 µM nebivolol, the density significantly decreased, while the area increased again, indicating disintegration of 92-1 spheroids (Figure 3A,C). In contrast, the area of UPMD2 and UPMM3 spheroids increased at ≥10 µM and decreased again at ≥30 µM (UPMD2) and ≥20 µM (UPMM3), respectively (Figure 3A,D,E). The density of spheroids of the primary cell lines was only slightly affected by nebivolol (Figure 3A,D,E). The density of UPMD2 spheroids was reduced at 10 µM but increased again at ≥15 µM, most likely due to a restructuring of the density distribution (Figure 3A,D). However, microscopic observation of UPMM3 spheroids at 20 µM exhibited a blurred spheroid shape, indicating disintegration of the outer cell layers. The latter observation was reflected by a decrease in UPMM3 spheroid density at ≥20 µM indicating lower compactness (Figure 3A,E). Taken together, the morphology of all spheroid types was affected by nebivolol treatment at ≥10–15 µM, suggesting inhibition of spheroid cell growth and 3D structure by nebivolol treatment.

**Figure 4:**
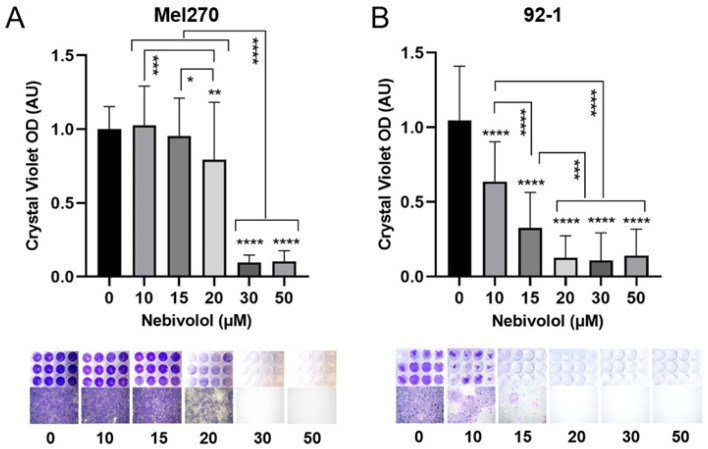
Long-term cytotoxicity of nebivolol-treated 3D tumor spheroid cells. At day 7, spheroids from cell lines (**A**) Mel270 or (**B**) 92-1 were treated with nebivolol at the indicated concentrations. Spheroid cultures were individually transferred to flat-bottom wells and cultured until control cells (0 µM nebivolol) were confluent. The long-term cytotoxic effect was determined in a spheroid cell survival assay. After 2 weeks in culture, the surviving cells were stained crystal violet (CV, OD 540 nm). Absorption of surviving cells was measured and normalized to the CV absorption of the control cells (0 µM nebivolol), given in arbitrary units (AU). Data are means ± SD of three independent experiments with n = 12 spheroids for each concentration. Statistical analysis was performed with one-way ANOVA and Tukey’s multiple comparisons test and significance levels are indicated at * *p* < 0.05, ** *p* < 0.01, *** *p* < 0.001, **** *p* < 0.0001. Representative images of CV staining of the spheroid-derived cell cultures (n = 12) and microscopic images of cells (10× magnification) for each concentration are shown below the graphs.

To confirm long-term effects of nebivolol treatment on the UM cells of our screening cell line Mel270 and the most nebivolol-responsive cell line, 92-1, we examined cell survival and repopulation ability of pretreated spheroids. After treatment, each spheroid was transferred individually to a flat-bottomed plate, allowing the remaining viable cells to grow out and repopulate (Figure 4). In cell line Mel270, complete tumor control was observed at concentrations of ≥30 µM nebivolol (Figure 4A). In the UM92-1 cell line, a response was evident at ≥10 µM. Complete blockade of tumor survival following treatment with nebivolol was observed at a concentration of 20 µM, thus preventing survival and repopulation of spreading spheroid cells (Figure 4B).

### 2.3. Comparison of Nebivolol Apoptotic Effects on Various 3D Tumor Spheroids

**Figure 5:**
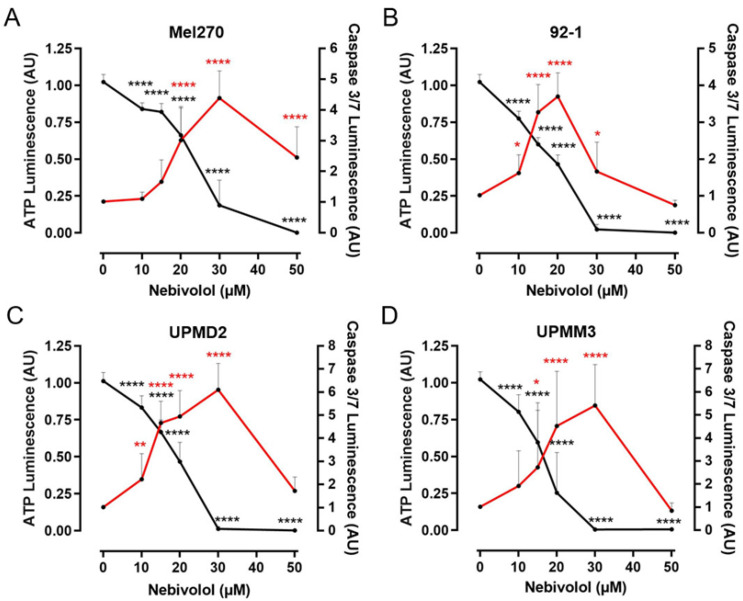
Early apoptotic effects and viability change of nebivolol in various 3D tumor spheroid models. Spheroids were generated from cell lines (**A**) Mel270 and (**B**) 92-1, or primary cell lines (**C**) UPMD2 and (**D**) UPMM3. Spheroids were treated once with nebivolol at the indicated concentration. Early apoptosis was assayed with a caspase 3/7 luminescence assay after incubation with nebivolol for 48 h (red line). Corresponding viability was assayed with an ATP luminescence assay (black line). The luminescence of the treated spheroids was normalized to the luminescence of the control spheroids (0 µM nebivolol) and is given in arbitrary units (AU). The means ± SD of three independent experiments for each UM tumor spheroid type with n = 6 spheroids for each condition are shown. Statistical analysis was conducted using one-way ANOVA and Tukey’s multiple comparisons test; the significance levels of the treated spheroids in relation to the control spheroids (0 µM nebivolol) are displayed. Significance levels are indicated at * *p* < 0.05, ** *p* < 0.01, **** *p* < 0.0001.

To investigate the early apoptotic effects of nebivolol in spheroids, caspase-3/7 activity was measured after 48 h (Figure 5). Caspase activity was induced by nebivolol in a concentration-dependent manner in all spheroid types. Caspase activity was significantly increased at 10 µM in 92-1 and UPMD2 spheroids, at 15 µM in UPMM3, and at 20 µM in Mel270 spheroids. Maximum caspase activity was measured at a nebivolol concentration of 20 µM in 92-1, while caspase activity was maximal at a concentration of 30 µM in all other spheroids (Figure 5B vs. Figure 5A,C,D). Correspondingly, ATP amounts in the spheroids were found to be already reduced by nebivolol at 10 µM. The ATP amount was completely depleted at 30 µM nebivolol in spheroid cell lines 92-1, UPMD2, and UPMM3, while the cell line MEL270 required a concentration of 50 µM nebivolol (Figure 5B–D vs. Figure 5A). In summary, Mel270 spheroids were revealed to be less sensitive to nebivolol treatment in comparison to the other cell lines. However, 92-1 spheroids turned out to be the most responsive, as early apoptosis was already maximal at 20 µM nebivolol. In the primary spheroid types, UPMD2 or UPMM3 apoptosis was maximal at 30 µM nebivolol (Figure 5B vs. Figure 5C,D).

### 2.4. Anti-Tumor Responses of Various 2D Cell Lines and Adrenergic Receptor Levels

**Figure 6:**
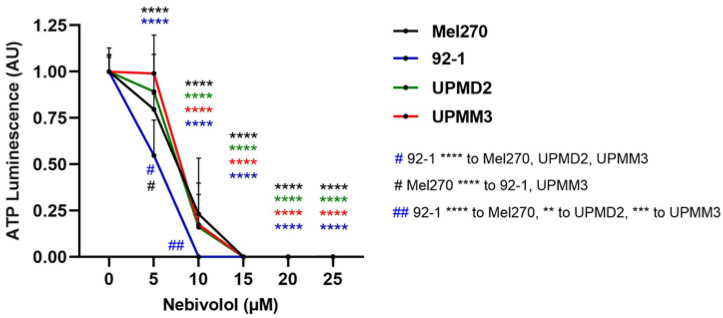
2D cell culture viability assay following the treatment with nebivolol. UM cell cultures Mel270, 92-1, UPMD2, and UPMM3 were treated once with nebivolol at the indicated concentration for 7 days. A cell viability ATP luminescence assay was performed, and the ATP luminescence of the treated cells was normalized to the ATP luminescence of the control cells (0 µM nebivolol), given in arbitrary units (AU). The means ± SD of three independent experiments for each UM cell line with n = 6–8 cultures each concentration are shown. Statistical analysis by two-way ANOVA and Tukey’s multiple comparisons test and the significance levels of the treated spheroids in relation to the control spheroids (0 µM nebivolol) are highlighted in color. Significance levels are indicated at **** *p* < 0.0001. The significance level of nebivolol treated cell lines compared to other cell lines is indicated by # or ## (figure legend bottom right corner ** *p* < 0.01, *** *p* < 0.001, **** *p* < 0.0001).

Additionally, the viability of the respective cell lines in response to nebivolol treatment was tested to determine whether the different sensitivity and responsiveness of the spheroids was due to the different 3D morphologies or whether cell-type dependent factors, such as genetics and proliferation rate, were involved (Figure 6). ATP was reduced concentration-dependently in all cell lines in the concentration range of 5–15 µM nebivolol in a comparable manner (Figure 6). However, the 92-1 cell line turned out to be most responsive to nebivolol when compared to the other cell lines (Figure 6). Thus, the responsiveness of cell lines was in line with their respective tumor spheroids (Figure 2), indicating that cell immanent factors were involved. However, the cell lines seemed to be more responsive than the 3D tumor spheroids (Figure 2).

**Figure 7:**
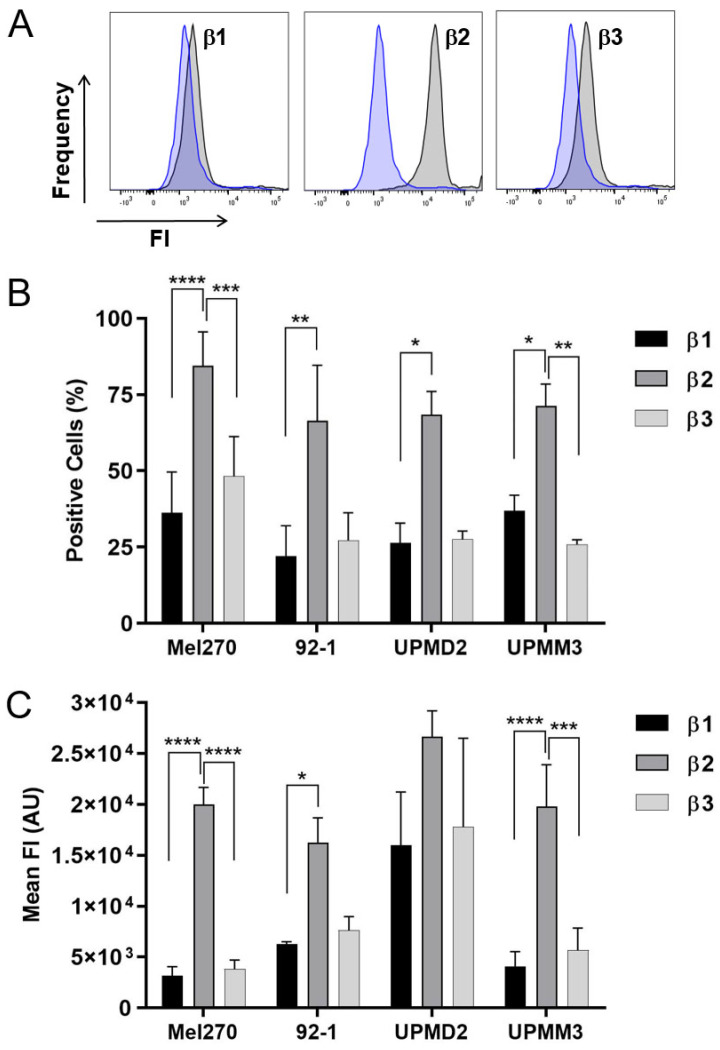
Presence of β-adrenergic receptors on the cell surface of UM cell lines. UM cell lines Mel270, 92-1, UPMD2, and UPMM3 were analyzed for the expression of β1-, β2-, or β3-adrenergic receptors (ADRB1, ADRB2, ADRB3) by flow cytometry. (**A**) Representative histograms of Mel270 cells stained for isotype control (blue) and the respective β-adrenergic receptors (grey). (**B**) Analysis of the frequency of cells positive for the respective β-adrenergic receptor given in (%). (**C**) Analysis of mean fluorescence intensities (FI) of stained cells given in arbitrary units (AU). Data represent the mean ± SD of at least two independent experiments for each cell line. Statistical analysis with two-way ANOVA and Tukey’s multiple comparisons test, significance levels were indicated at *p* values: * < 0.05, ** < 0.01; *** < 0.001, **** < 0.0001.

Next, it was examined whether the nebivolol sensitivity of UM cells was related to varying ß-adrenergic receptor levels (Figure 7). Therefore, we analyzed the presence of ß1, ß2, and ß3-adrenergic receptors on cell surfaces by flow cytometry (Figure 7A). The ß-adrenergic receptors were expressed in all cell lines and approximately 70–90% of UM cell populations were positive for β2 receptors, while 25–50% of cells were positive for ß1 or ß3 adrenergic receptors. Among the cell lines, Mel270 presented the highest percentage of positive cells (Figure 7B). Analysis of the mean fluorescence intensity of the stained cells revealed that the β2-adrenergic receptor was most strongly expressed in all cell lines (Figure 7C). However, UPMD2 cells showed large variations in β-adrenergic receptor expression levels. Conclusively, the data suggest that the β2-adrenergic receptor is most prominently expressed on the cell surface of UM cells.

### 2.5. Effects of a Highly Selective ß2-Antagonist and Discrimination between the Different Nebivolol Enantiomers

**Figure 8:**
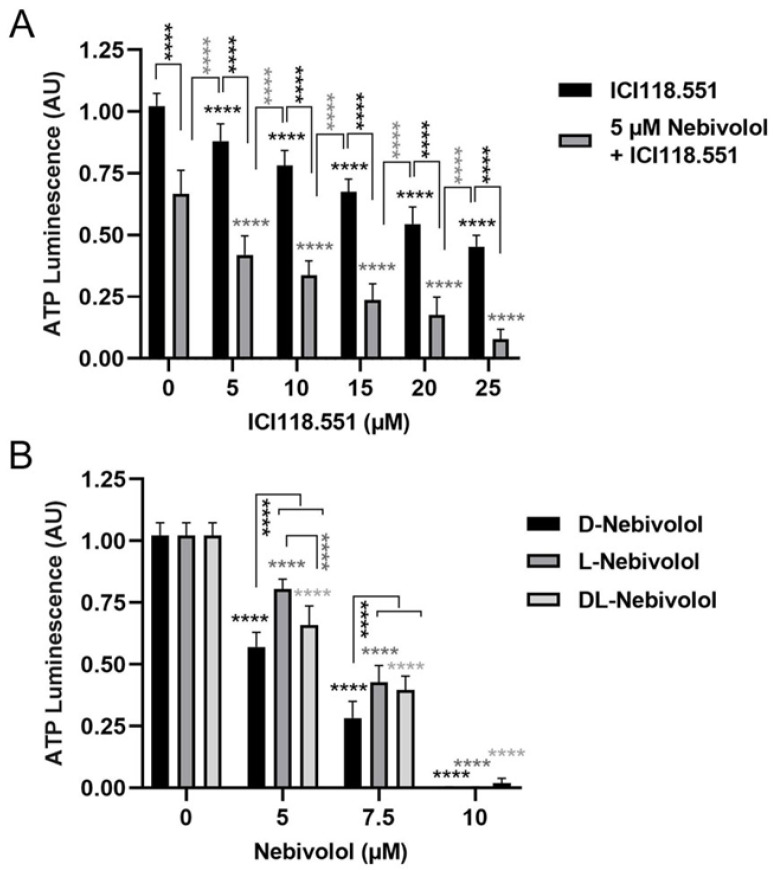
Effects of β2-adrenoceptor antagonism and of nebivolol enantiomers on ATP levels of Mel270 cells. (**A**) Cells were treated once with β2-antagonist ICI 118.551 at the indicated concentrations in the absence or presence of 5 µM nebivolol. (**B**) Cells were treated once with either D-nebivolol, L-nebivolol, or racemic DL-nebivolol at the indicated concentrations. After 7 days of incubation, cell ATP was assayed with an ATP luminescence assay. ATP luminescence of treated spheroids was normalized to ATP luminescence of controls (0 µM) and is given in arbitrary units (AU). The means ± SD of three independent experiments with n = 6 cultures each condition shown. Statistical analysis by two-way ANOVA and Tukey’s multiple comparisons test, significance levels were indicated **** *p* < 0.0001.

Nebivolol is a β1-selective blocker that also binds to β2-receptors; however, the latter is with lower affinity [14,22]. To explore the involvement of β2-receptors in the anti-tumor mechanism, we tested the highly selective β2-antagonist ICI 118.551 alone or in the presence of nebivolol on Mel270 cells (Figure 8). The β2-antagonist diminished ATP levels in a concentration-dependent manner at 5 to 25 µM; however, it was less efficient when compared with nebivolol, as it showed no complete ATP reduction, tested up to 25 µM (Figure 8A). Moreover, in combination with 5 µM nebivolol, the β2-antagonist exhibited an additional reduction of ATP levels. Hence, the data indicate that antagonism of β2-receptors can elicit anti-tumor activity in UM cells.

However, the clinically used nebivolol is a racemic mixture consisting of D- and L-enantiomers, with D-nebivolol showing the highest β1-receptor affinity [17,22,44]. We therefore tested whether an isolated enantiomer would have an even better inhibitory effect on UM cell viability than the racemic DL-nebivolol. We found that both enantiomers significantly diminished the ATP levels of Mel270 cells at a concentration range of 5–10 µM. Of note, D-nebivolol reduced ATP-levels most efficiently compared with L-nebivolol or DL-nebivolol, implicating that β1-blocking is involved in the reduction of ATP levels and thus viability of UM cells (Figure 8B).

## 3. Discussion

The purpose of this study was to investigate the anti-tumor effects of different β1-selective blockers on UM cell lines. A concentration-dependent reduction in ATP level and, thus, cell viability was documented following treatment with nebivolol only, while all other β1-blockers tested, atenolol, celiprolol, bisoprolol, metoprolol, esmolol, and betaxolol, showed no anti-tumor effect in 3D tumor spheroids (Figure 1). Therefore, the subsequent experiments were conducted with nebivolol, demonstrating a significant change in 3D structure of the tumor spheroids as well as ATP depletion, depending on the cell line investigated (Figure 2 and Figure 3). Furthermore, an upregulated apoptosis and, finally, complete tumor control in a long-term survival assay was demonstrated at a concentration of ≥20 µM (Figure 4 and Figure 5). The usage of 3D tumor spheroids as a cell model conferred a higher level of evidence to our study, as spheroids are known to mimic in vivo tumors in their 3D morphology by enabling cell–cell interactions and maintaining a microenvironment similar to the one found in the tumor. This also leads to increased comparability of drug penetration due to their similar architecture, e.g., the inner necrotic zone, and thus may be accountable for higher resistance to drug treatment compared to monolayer 2D cell models [45,46,47,48]. In line with this, UM 3D tumor spheroids required a higher concentration of nebivolol for complete blockade of viability (20-30 µM), than the respective UM cell lines (10–15 µM) (Figure 2 and Figure 6). We observed cell type-dependent responsiveness of spheroids, suggesting that cell type immanent factors play a role rather than differences in spheroid structures (Figure 2, Figure 3, Figure 4, Figure 5 and Figure 6). Cell type-immanent factors, such as genetic profiles and cell morphology, have been frequently attributed to therapeutic outcomes in UM [25,30,36,37,38,39]. However, in our study, the presence of β-adrenoceptors on cell surfaces was required for the anti-tumor effect of ß-blockers, and differences in expression levels may have accounted for cell responsiveness.

Consistently, all UM cell lines expressed the three β-receptors on cell surfaces, predominantly β2-receptors (Figure 7). Among the cell lines, Mel270 population constitutes the highest proportions of β-receptor positive cells (Figure 7), which may account for higher concentrations of nebivolol for the blocking of viability or long-time survival of spheroid cells (Figure 2, Figure 3, Figure 4, Figure 5 and Figure 6). Interestingly, Mel270 spheroid cells showed tumor-controlling potential of nebivolol at the same concentration as the non-selective β-blocker carvedilol in our previous study (Figure 4), [41]. Concerning the cell sensitivity in the viability assay, however, the anti-tumor efficiency of nebivolol for the various spheroid types differed from carvedilol. The 92-1 cells were more responsive to nebivolol than to carvedilol, although nebivolol is known to have a lower binding affinity to β2-receptors than carvedilol [22], implicating that β1-receptors are involved in the anti-tumor effects of nebivolol. However, among the β1-selective blockers tested in the present study, nebivolol exerts the highest binding affinity not only to β1-receptors but also to β2-receptors [22], thus combined β1/ β2-binding affinities may account for the exclusive anti-tumor activity of nebivolol among the ß1-selective blockers tested (Figure 1). Consistently, the highly selective β2-antagonist ICI 118.551 [22] decreased viability of Mel270 cells when applied alone or additive to nebivolol, suggesting that anti-tumor effects can be achieved by blocking the β1- and β2-receptors of UM cells (Figure 8). However, both nebivolol and carvedilol were highly efficient in blocking the viability of UPMM3 tumor spheroids (Figure 2), [41]. This is of importance as the UPMM3 cell line represents a more aggressive tumor type with high metastasis potential due to its monosomy-3 genetic profile, and recent studies have described a higher resistance of UM cells containing monosomy-3 to other therapeutics, e.g., MEK inhibitors [30,36,37,49].

In the present study, we measured ATP levels of spheroids as an indicator of cell viability. The reduction of ATP levels following the treatment with nebivolol may have been influenced by the inhibition of the proliferation and thus a lower cell number after a 7-day incubation period. Consistent with this, a pre-treatment with nebivolol reduced repopulation of tumor cells in a long-time survival assay, indicating a reduction in proliferation capacity (Figure 4). However, we were able to detect complete depletion of ATP levels even in the slowly proliferating UPMM3 and UPMD2 cells after an incubation time of only 48 h (Figure 5), indicating a direct effect of nebivolol on ATP homeostasis in the cells rather than a decreased cell number. In fact, recent studies attributed the anti-tumor activity of nebivolol to the inhibition of mitochondrial activity [24,50]. Nebivolol inhibited mitochondrial respiration and ATP synthesis, leading to ATP depletion in colon and breast cancer cells [50]. Mitochondrial respiration was affected neither by the ß2-receptor antagonist ICI 118.551 nor by the β3-receptor antagonist SR 59230A. Furthermore, the study excluded a direct effect of nebivolol on isolated mitochondria [50]. These results emphasize that ß1-receptor-dependent signaling is involved in the inhibition of mitochondrial activity and thus ATP depletion by nebivolol in cancer cells.

In addition, we found the activity of the cell death executioner caspase-3/7 progressively induced in 3D tumor spheroids, indicating that apoptotic signaling events have been initiated concentration-dependently by nebivolol (Figure 5). However, caspase-3/7 activity decelerated in response to higher nebivolol concentrations ≥20–30 µM. At these concentrations, ATP levels were already totally depleted and most likely limited caspase-3/7 activity (Figure 5). It is well known that activation of caspase-3/7 requires the processing of pro-caspase-3, which is dependent on cytochrome c release from impaired mitochondria, other factors, and ATP. Subsequently, caspase-3/7 activity triggers apoptosis and finally cell death [51,52,53]. Consistent with this, nebivolol concentrations ≥20–30 µM completely blocked spheroid cell repopulation, allowing tumor control (Figure 4). Taken together, both ATP depletion and induction of caspase-3/7 activity suggest that mitochondria are affected by nebivolol treatment of UM cells. The signaling events downstream of β-receptor blockade that might target mitochondrial activity in UM cells need to be further elucidated.

Furthermore, the clinically used nebivolol is a racemic mixture consisting of two enantiomers with different activities [17,22,44]. Since nebivolol may possess numerous mechanisms, such as activation of eNOS [22], besides the blockade of β1- or β2-receptors, which have not been adequately investigated to date, we have attempted to further narrow down the anti-tumor effect of nebivolol on UM cells. Although there is a lack of studies distinguishing the two different enantiomers, D-nebivolol was shown to have a 175-fold higher binding affinity to β1-adrenergic receptors than L-nebivolol [22,44]. In our study, the isolate D-nebivolol revealed increased potency in a viability assay compared to L-nebivolol or DL-nebivolol (Figure 8B). The data suggesting a stronger effect of D-enantiomer are consistent with an involvement of β1-receptors in the anti-tumor mechanism of nebivolol. However, unlike nebivolol, D-nebivolol is not FDA-approved, and thus cannot be freely repurposed for cancer therapy.

In conclusion, nebivolol is the most potent anti-tumor β1-blocker for UM identified in our studies using 3D tumor spheroids or cell lines to date. UM cells expressed all three β-adrenergic receptors on the cell surfaces, predominantly β2-receptors. Our results suggest that the combined binding affinities for β1/ β2-receptors are involved in its anti-tumor activities. Nebivolol induced ATP depletion and caspase-3/7 activity, which led to cell death. Our preclinical data indicate that certain β-blockers such as nebivolol may be suitable for co-adjuvant treatment of UM to support local tumor control and to prevent recurrence or metastasis. Although nebivolol is an already FDA-approved drug, the tumor control potential of nebivolol for UM needs to be further assessed by subsequent in vivo studies using orthotopic mouse models or the chicken chorioallantoic membrane assay, which we have recently optimized for UM 3D tumor spheroids [54].

## 4. Materials and Methods

### 4.1. Characteristics of Cell Lines

To obtain representative results in this study, four different UM cell lines were used, consisting of different genetic profiles and cell characteristics, which may influence treatment responses (Table 1). The UM cells were obtained from untreated primary tumors, except Mel270, which originated from an irradiated tumor (Table 1). Short tandem repeat profiling was performed for all cell lines, according to published data [43,55]. The different cell lines lead to different 3D tumor spheroids as described in detail before [42].

### 4.2. Culture of Cell Lines and 3D Tumor Spheroids

For maintaining Mel270 and 92-1 cell lines, RPMI 1640 medium (GIBCO, Fisher Scientific, Thermo Fisher Scientific Inc., Waltham, MA, USA) was used, supplemented with 1% penicillin–streptomycin (5000 U/mL, PAN BIOTECH, Aidenbach, Germany) as well as 10% fetal calf serum (Sigma-Aldrich, St. Louis, MO, USA/Chemie GmbH, Steinheim, Germany). UPMD2 and UPMM3 were kept in Hams/F12 medium (PAN-Biotech GmbH, Aidenbach, Germany) and the respective supplements. The medium was renewed twice weekly. The cell lines were incubated in a humidified incubator (37 °C, 5% CO_2_).

3D tumor spheroids were grown in round-bottom 96-well ultra-low attachment plates (PHC Corporation, Tokyo, Japan) using 5 × 10^3^ living cells and 100 µL of the respective cell culture medium per well. Spheroids were grown for 7 days, while medium was refreshed once weekly, as described before [42].

### 4.3. Treatment with β-Blockers

The following β1-selective adrenergic receptor-blockers from Sigma-Aldrich (St. Louis, USA/Chemie GmbH, Steinheim, GE) were used in this study: atenolol (CAS No.: 29122-68-7), celiprolol hydrochloride (CAS No.: 57470-78-7), bisoprolol (CAS No.: 66722-44-9), metoprolol tartrate (CAS No.: 56392-17-7), esmolol hydrochloride (CAS No.: 81161-17-3), betaxolol-hydrochloride (CAS No.: 63659-19-8), and nebivolol hydrochloride (CAS No: 152520-56-4). In other experiments, enantiomers of nebivolol D-(+)-nebivolol (CAS No.:118457-15-1), L-(-)-nebivolol (CAS No.: 118457-16-2), and racemic nebivolol (DL-nebivolol) (CAS No.: 99200-09-6) were purchased from Cymit Quimica S.L. (Barcelona, Spain). The β2-selective adrenergic receptor antagonist ICI118.551 hydrochloride (CAS No.:72795-19-8) was obtained from Sigma–Aldrich. Stock solutions of 50 mM β-blocker were prepared in dimethyl sulfoxide (DMSO) and frozen until needed. The spheroids and the 2D cell cultures were treated once and incubated in the respective medium for the indicated time period. Controls (0 µM β-blocker) received DMSO at the same concentration as the highest concentration of the respective β-blocker, no effect of solely DMSO treatment on the spheroids or cells was measured.

### 4.4. 3D Spheroid Viability Measurement

Changes in cellular ATP levels in tumor spheroids (n = 6–8 spheroids for each β1-blocker concentration) were determined by the usage of the CellTiter-Glo 3D Cell Viability assay (Promega GmbH, Walldorf, Germany). Luminescence was detected with the FluostarOmega reader (BMG LABTECH, Ortenberg, Germany). The procedure was as follows: an equal amount of Cell-Titer Glo 3D reagent (100 µL) and the treated spheroid culture was mixed by pipetting up and down for 30 s. The mixture was transferred to a white opaque-walled multi-well plate (Nunc, Thermo Fisher Scientific, Roskilde, Denmark), incubated for five minutes on a shaker at 750 rpm, followed by further 25 min under light protection. The ATP luminescence (relative light units) of the spheroids was normalized to the ATP luminescence of the control spheroids and is given in arbitrary units (AU).

### 4.5. Analysis of 3D Spheroid Apoptosis

Caspase-3/7 activity in tumor spheroids (n = 6 spheroids each nebivolol concentration) was analyzed with Caspase-Glo 3/7 assay (Promega GmbH, Walldorf, Germany). Equal amounts of substrate solution and tumor spheroid culture were mixed 30 s by pipetting up and down and measured in a white opaque-walled multi-well plate (Nunc, Thermo Fisher Scientific, Roskilde, Denmark) using the reader FluostarOmega (BMG LABTECH, Ortenberg, Germany). The mixture was incubated for 30 s on a shaker at 500 rpm and further incubated in the dark for 40 min. The caspase-3/7 luminescence (relative light units) of treated spheroids was normalized to the control spheroids and is given in arbitrary units (AU).

### 4.6. Morphology, Growth, and Density Determination of 3D Spheroids

The spheroid cultures (n = 5 spheroids each nebivolol concentration) of the four different cell lines were treated with nebivolol on day 7. Imaging was conducted with a Zeiss Primovert bright-field microscope at 4× magnification and a Zeiss Axiocam 105 and ZENcore software on day 7 and day 14. Images were then analyzed with ImageJ Fiji (MPI-CBG, Dresden, Germany). The size and compactness of the treated spheroids were calculated by the measurement of the cross-sectional area of spheroids (µm^2^) and optical density of the spheroid area (mean grey value). The cross-sectional area and density of the treated spheroids were normalized to the mean of the spheroids before treatment (day 7) and are given in arbitrary units (AU).

### 4.7. Long-Term Spheroid Cell Survival Assay

After an incubation period of four days following treatment with nebivolol, the tumor spheroids (n = 12 per condition) were transferred individually into 24-well flat-bottom plates (Cellstar, Greiner Bio-One GmbH, Frickenhausen, Germany) to allow outgrowth of surviving cells. Cells were cultured until the control cells were 90% confluent, allowing surviving cells to repopulate. Medium was refreshed two times a week. Adherent cells were fixated with 4% formaldehyde for 5 min and stained with 0.05% crystal violet (CV) for 15 min. The absorbance of the stained nuclei/DNA was detected with a reader ClarioStar Plus (BMG LABTECH, Ortenberg, Germany) at OD 540 nm. The CV absorbance (relative absorbance units) of the treated spheroids was normalized to the CV absorbance of the control cultures and is given in arbitrary units (AU). Zeiss Primovert’s bright-field microscope at 10× magnification was used to document CV-stained cultures. Images were recorded with a Zeiss Axiocam 105 and ZENcore software.

### 4.8. Cell Viability Analysis in 2D Cell Cultures

2D experiments were performed in flat-bottom 96-well plates (Sarstedt, Nümbrecht, Germany); 5 × 10^3^ cells per well were seeded overnight. Cell cultures were treated with nebivolol for 7 days (n = 8 each concentration) and then subjected to CellTiter-Glo 2D viability assay (Promega GmbH, Walldorf, Germany). An equal volume of the CellTiter-Glo reagent and of the cell cultures were mixed by pipetting up and down for 10 s. The mixture was recorded using a reader FluostarOmega (BMG LABTECH, Ortenberg, Germany) in white opaque-walled multi-well plates (Nunc, Thermo Fisher Scientific, Roskilde, Denmark) with a prior incubation time of two minutes on a shaker at 750 rpm and a further 10 min incubation under light protection. The ATP luminescence (relative light units) of the treated spheroids was normalized to the ATP luminescence of the control cells and is given in arbitrary units (AU).

### 4.9. Detection of β-Adrenergic Receptors by Flow Cytometry

For the detection of β-adrenergic receptors on cell surfaces, 5 × 10^5^ UM cells were incubated with rabbit anti-human ADRB1, ADRB2, or ADRB3 antibodies (Bioss Antibodies Cat. No. bs-0498R, bs-0947R, bs-1063R), or rabbit IgG control (Dako X 0903) in cell staining buffer (1% calf serum albumin and 0.1% NaN3 sodium azide in PBS for 30 min at 4 °C). After two washing steps with cell staining buffer, the cells were incubated with the secondary antibody goat anti-rabbit Alexa Fluor 488 (Invitrogen Cat. No. A11008, Walthman, MA, USA) for 30 min at 4 °C. After three washing steps with cell staining buffer, the cells were fixed with 4% paraformaldehyde for 15 min at 4 °C, finally resuspended in PBS and stored at 4 °C. Each cell line 10^4^ cells were analyzed by BD FACS Aria III. Histograms of cell populations were visualized and analyzed by usingFlowJo-v10.8.1.

### 4.10. Statistical Analysis

Statistical analysis of the data was performed using GraphPad Prism (GraphPad Prism 8.4.3 software, GraphPad Software Inc., San Diego, CA, USA). Data were analyzed by one-way ANOVA or two-way ANOVA and Tukey’s multiple comparisons test. Statistical significance was considered at a value of *p* < 0.05. The significance levels indicated are as follows: * *p* < 0.05, ** *p* < 0.01; *** *p* < 0.001, **** *p* < 0.0001.

## Figures and Tables

**Table 1:** Characteristic of uveal melanoma cell lines.

Cell Line	Genetics	Morphology/Doubling Time	Prior Therapy	References
Mel270	GNAQ Q209P, disomy-3	Spindle/43 h	Irradiation	[43,56,57]
92-1	GNAQ Q209L, disomy-3, EIF1AX mutant	Epithelioid/38–58 h	Untreated	[43,57,58,59]
UPMD2	GNA11 Q209L, disomy-3	Epithelioid/150 h	Untreated	[49,55,60]
UPMM3	GNAQ Q209P, monosomy-3, BAP1 mutant	Spindle and Epithelioid/100 h	Untreated	[49,55,60]

## Data Availability

Not applicable.
